# Prospective mixed-methods study evaluating the potential of a voicebot (CovBot) to relieve German health authorities during the COVID-19 infodemic

**DOI:** 10.1177/20552076231180677

**Published:** 2023-06-07

**Authors:** Vanessa Voelskow, Claudia Meßner, Tobias Kurth, Amelie Busam, Toivo Glatz, Natalie Ebert

**Affiliations:** Institute of Public Health at Charité – 14903 Universitätsmedizin Berlin, Berlin, Germany

**Keywords:** Voicebot, COVID-19, evaluation, conversational hotline agents, conversational technologies, pandemics, infodemic, hotline, digital health, public health

## Abstract

**Background:**

During the COVID-19 pandemic, telephone hotlines of local health authorities in Germany were overloaded due to information requests by the public.

**Objective:**

Evaluating the use of a COVID-19-specific voicebot (CovBot) in local health authorities in Germany during the COVID-19 pandemic. This study investigates the performance of the CovBot by assessing a perceptible relief of staff in the hotline service.

**Methods:**

This prospective mixed-methods study enrolled local health authorities in Germany from 01 February 2021 to 11 February 2022 to deploy the CovBot, which was mainly designed to answer frequently asked questions. To capture the user perspective and acceptance, we performed semistructured interviews and online surveys with their staff, conducted an online survey among callers, and analyzed the performance metrics of the CovBot.

**Results:**

The CovBot was implemented in 20 local health authorities serving 6.1 million German citizens and processed almost 1.2 million calls during the study period. The overall assessment was that the CovBot contributed to a perceived relief of the hotline service. In a survey among callers, 79% indicated that a voicebot could not replace a human. The analyzed anonymous metadata revealed that 15% of calls hung up immediately, 32% after hearing an FAQ answer, and 51% of calls were forwarded to the local health authority offices.

**Conclusions:**

A voicebot primarily answering FAQs can provide additional support to relieve the hotline service of local health authorities in Germany during the COVID-19 pandemic. For complex concerns, a forwarding option to a human proved to be an essential functionality.

## Introduction

### Background

The COVID-19 pandemic was accompanied by an overabundance of ever-changing information. For the general public, it was unclear which sources were up-to-date or trustworthy.^1^ Health information systems were overwhelmed, and—due to the urgency of the situation—the adaptation lacked proper strategic planning, frequently resulting in inaccurate or outdated information due to implementation delays.^2^ In addition to the necessary infection prevention measures, the World Health Organization (WHO) called for a coordinated, evidence-based infodemic management.^3,4^

In Germany, around 400 local health authorities played a key role in providing information to the public during the COVID-19 pandemic. These offices with specially trained staff served as the first point of contact for infection hygiene measures to contain the pandemic. Each day, health offices received a large number of virtually identical inquiries (e.g. where to get tested, how to proceed when testing positive, or what quarantine regulations hold in a given municipality),^3,5,6^ which led to an overload of telephone hotlines early on in the pandemic.^7^ Identifying ways to support the hotline service of local health authorities for contributing to a successful pandemic response was therefore of utmost importance.

Technologies that imitate natural conversations, such as voicebots or chatbots, present an opportunity to aid in the dissemination of information. Both voicebots and chatbots are programmed to answer frequently asked questions (FAQs) with predefined answers for common concerns.^8^ However, the communication modalities differ between voicebots and chatbots: voicebots rely on speech recognition and synthesis to mimic a telephone conversation, whereas chatbots use a text-based messenger design. Recent studies have indicated the potential benefits of automated telephone communication to answer health-related questions, while also identifying the need for continued scientific evaluation of the use of these novel systems in the healthcare sector. In particular, the user perspective and acceptance are yet to be comprehensively investigated, as only a few studies currently include these aspects.^9,10^

Against this background, we investigated the use of a hotline assistant (**co**rona**v**irus voice**bot** or CovBot) using artificial intelligence (AI)^11^ to assist local health authorities. The contracted company's responsibility involved implementing the CovBot and its further development according to several needs analyses conducted with the local health authority offices. The starting point was the adaptation of an existing hotline assistant for physician's offices^7,8^ to COVID-19-related questions.

### Study objective

The goal of the present study was to perform a broad evaluation of the use of a voicebot by local health authorities in Germany during the COVID-19 pandemic. A prospective mixed-methods design based on a prior needs analysis and pilot study was applied. This included interviews and surveys among staff and callers supplemented by statistical analyses of available metadata (e.g. proportion of hung-up calls after hearing an FAQ answer or calls forwarded to a staff member). We evaluated the performance of the CovBot with regard to acceptance among callers and staff, as well as a perceptible relief of staff in the hotline service, and performance metrics based on call metadata as the most relevant outcomes. We expected that a combination of all these measures would provide valuable insights into the use of an automated telephone communication assistant during the COVID-19 pandemic. A better understanding of contextual factors influencing the implementation of the rapidly developing novel technology of AI-based bots, including theories for their acceptance^12–14^ can be achieved with hypothesis-based data generation. This is particularly important in the domain of public health.

## Methods

### Needs analysis and pilot study

Prior to the main study, we performed a needs analysis followed by a pilot study from 13 April 2020 to 31 January 2021. An anonymous online survey was first sent via e-mail to all local health authorities in Germany to assess their needs for a voicebot in general.^15^ Based on these results, we selected the most relevant functionality for a first implementation with the industry partner Aaron GmbH^11^ of the CovBot, and then piloted it in three local health authorities. We conducted both exploratory interviews with the head of each local health authority and surveys among staff in the hotline service. The CovBot thereby received an initial, yet preliminary, appraisal for its use under real-world conditions.

### Main study

Study design: This study was a prospective exploratory non-controlled mixed-methods study designed as an emergency response to evaluate the potential of a voicebot to relieve overwhelmed German local health authority hotlines during the COVID-19 infodemic.

### Inclusion criteria

During the main study period from 01 February 2021 to 11 February 2022, we intended to recruit up to 20 local health authorities in Germany. In order to obtain a representative sample of Germany's diverse landscape of local health authorities, we applied a covariate adaptive inclusion process (i.e. minimization) with consideration of the strata: geographical region (north/east/south/west), infrastructure (urban/rural), district administration (yes/no), and size (small/medium/large). The area of responsibility was determined based on the number of citizens which each local health authority served as indicated by the local health authorities (e.g. on their webpages). Those with less than 130,000 inhabitants were categorized as “small,” with at least 130,000 and less than 350,000 inhabitants as “medium,” and with at least 350,000 inhabitants as “large.” While we did not have any specific exclusion criteria, we only enrolled a maximum of seven local health authorities per stratum of region and size.

### Recruitment

For recruitment, 120 informational invitation letters were sent by post to managers of local health authorities, a call for participation was published on the website of the Institute of Public Health at Charité—Universitätsmedizin Berlin^16^ and subject-specific online events were used to actively promote the study.^6,17,18^

### Mixed-methods evaluation

The primary focus of this study was to assess the performance of the CovBot with regard to a perceptible relief of staff in the hotline service. Further exploratory research questions addressed the duration of the implementation phase and potential use cases beyond the pandemic. In addition, a product-specific needs analysis was conducted for the further development of the CovBot.

#### Qualitative evaluation

The qualitative part of the study consisted of semistructured, guideline-based expert interviews^19^ of up to 30 minutes. We conducted telephone interviews with a manager from each local health authority and an employee from either the hotline service or the information technology (IT) department after six to eight weeks of actively using the voicebot. All participants provided written informed consent. Interviews were conducted by two researchers and were not recorded. During the interview, one researcher was responsible for leading the exchange while the other simultaneously created a pseudonymized transcript of the interview. Afterward, the transcript was sent to the participants for possible feedback. The data were analyzed according to the qualitative content analysis^20^ with the software MAXQDA 2020.^21,22^

#### Quantitative evaluation

Employees in the hotline service were asked to complete anonymous online questionnaires two to three days before the implementation of the voicebot (predeployment questionnaire with 18 items using yes/no and 5-point Likert scale answers) and two to three months after (postdeployment questionnaire with 38 items using yes/no, 5-point Likert scale and free text answers). The user perspective was captured by an anonymous survey among callers (≥ 18 years). The voicebot thereby automatically invited every 20th caller to participate before the dialog, limited to 10 participation requests per day per local health authority. If callers agreed to participate, they got a link sent by SMS message (SMS survey) to fill out an online questionnaire (18 items using yes/no, 5-point Likert scale and free-text answers). The caller's phone number was automatically and irrevocably deleted by the voicebot immediately after the SMS message was sent.

We assessed the anonymized metadata of the CovBot by descriptively analyzing: (1) the total number of calls per local health authority over the study period, (2) the proportion of calls that were hung up before the CovBot could ask for the intent, (3) the proportion of recognized and unrecognized intents, (4) the total number of FAQ answers provided, (5) the total number of calls that were hung up after listening to an FAQ answer, (6) the proportion of calls in which the caller wanted to be forwarded to a staff member, and (7) the proportion of calls that was successfully forwarded to a staff member. These metrics were captured over the entire study period by Aaron GmbH. In accordance with data protection laws, no information that could identify a caller (including, but not limited to, a transcription of what the caller said or their phone number) was stored. The anonymous and secure use of the CovBot according to data protection regulations was an utmost priority for all involved project partners. Analyses were performed descriptively using R 4.1.3.^23^

To assess the CovBot's recognition of predefined intents and its robustness to unexpected inputs we further conducted test calls at the end of the study phase in February 2022.^24^ Based on the probability distribution of the nine most common intents (corresponding to 94% of all recognized intents) and the calls where no predefined intent was recognized in the real-world metadata, we created a list of 300 intents for test calls. Of these, 191 assessed predefined intents and 109 unknown (i.e. not predefined) intents. Fifteen callers (eight female; age range 20–50; all German native speakers), of whom five were project staff and 10 were naive callers who did not know the bot previously, were asked to make 20 calls in a prespecified order and come up with a realistic prompt for each given endpoint (e.g. by saying “ I had contact with a person who tested positive for covid” to reach the predefined intent “COVID-19 contact,” or by saying “I would like to inquire about the school entry health exam for my child,” which health authorities also offer, but which is not part of the CovBot). We report the proportions of correctly recognized predefined intents and correctly ignored unconfigured intents.

The descriptive needs analysis for further development of the CovBot in accordance with the requirements set forth by the local health authorities during the pandemic included both qualitative and quantitative components. We first collected suggestions for further development of the CovBot from the managers’ interviews and staff questionnaires, but also suggestions from regular (weekly/monthly) unstructured feedback meetings with the project participants in the respective local health authorities. The results were prioritized in a survey by employees in management functions (18 items using 5-point Likert scale and free-text answers). All study procedures were developed in line with our study objectives and approved by the ethics committee of the Charité—Universitätsmedizin Berlin (EA4/191/20, EA1/211/21). We tested the interview forms and surveys during the pilot phase of the study and adapted them for the main period based on the gained experiences (see Supplemental Material).

For better readability, results are presented in chronological order (i.e. beginning with the implementation) with the respective qualitative and quantitative results, if applicable, presented one after the other for each aspect.

## Results

### Needs analysis and pilot study

From 3 June to 22 June 2020, the pilot needs survey was completed by 102 staff members of local health authorities throughout Germany. It showed a general interest in relief from additional tasks that had arisen in the hotline service during the COVID-19 pandemic. The pilot study included the subsequent implementation of the CovBot in three local health authorities in Germany, whereby a total of 114,842 calls were processed between 3 September 2020 and 31 January 2021. Hotline service employees indicated a perceived reduction in the number of calls containing FAQs during the pilot phase. The interviewed heads of the local health authorities for the pilot study, stated that the satisfaction and acceptance among staff members with the CovBot were good. There were no relevant complaints of callers reported, although the caller's perspective was not formally assessed during the pilot study.

#### The CovBot—description of the assessed voicebot

We continued the collaboration with Aaron GmbH to implement and further develop the CovBot for the main study. By that time, the CovBot mainly possessed the functionality to recognize preconfigured concerns in natural speech (i.e. Automatic Speech Recognition for speech-to-text conversion) and reply with predefined FAQ answers (i.e. text-to-speech synthesis). Due to the urgency of the pandemic, the aim was to achieve a timely deployment of the voicebot and allow for full future flexibility regarding the configured intents in compliance with German data protection regulations. The need for rapid deployment made it impossible to collect extensive training data. Therefore, a hybrid approach was taken for intent recognition. By default, the CovBot used traditional computational methods based on the occurrence of keywords. If these did not yield results, they were supplemented with pretrained machine learning algorithms. The machine learning algorithms for the text classification tasks relied on models built by Aaron GmbH. They were based on the random forest algorithm^25^ and trained using both existing and newly generated proprietary datasets. The use of zero-shot learning techniques was initially intended but turned out not to be feasible due to time constraints. Aaron GmbH used pretrained machine learning models of Google Cloud for the speech recognition^26^ and speech synthesis^27^ using a female voice.

The assessed version of the CovBot from June 2021 could answer a single concern per call. As the main intention was to reduce call volume, all callers were first connected to the CovBot irrespective of staff availability. When there was either no input, or the CovBot could not recognize the intent of the caller, the caller was asked to repeat their concern. If no predefined intent was recognized for the second time or the caller felt that the answer of the CovBot for a recognized intent was not sufficient, there was a forwarding option to an employee that local health authorities could switch on and off manually. If activated, the CovBot forwarded calls to predefined phone numbers depending on the recognized intent. While some local health authorities assigned phone numbers to individual staff members, there was an option to install automatic call distribution features for larger local health authorities. Besides the forwarding option, there was an optional (i.e. configurable by the health offices) voice messaging function asking callers to leave a voice message, which would be subsequently transcribed to facilitate callbacks in a so-called WebApplication (WebApp). These transcriptions were only visible to the health authority and not part of the study data collection. Once the voice message functionality was activated, the WebApp displayed a basic statistic about the call volume of the previous day. During the regular feedback meetings, Aaron GmbH presented statistics on the proportion of calls forwarded to an employee in relation to all incoming calls. The working routine for the staff in the hotline service remained almost unchanged. Further technical details are beyond the scope of this article.

During the pilot and first study phase, each local health authority developed individual predefined FAQ answers. Aaron GmbH provided best-practice guidelines and support if needed. To optimize the CovBot implementation in local health authorities, a so-called “self-service configuration” was made available in June 2021. This enabled the local health authorities to access predefined FAQ answers for the most frequent concerns provided by Aaron GmbH. They were based on responses developed by other local health authorities during the pilot and first study phase when self-service configuration was not yet installed. The local health authorities could independently configure and change these FAQ texts. Fourteen health authorities engaged in this process. This feature was subsequently available for the local health authorities using the CovBot at that time, and as of July 2021, all participating local health authorities used the CovBot with the WebApp. Issues surrounding the accessibility of the voicebot for staff and callers alike were also considered during the design of the WebApp. The addition of dialog templates and tooltips with recommendations on accessible language made it easier for the local health authorities to create FAQ answers in an easy-to-comprehend language. Aaron GmbH also added controls for speaking rate, pauses, and pronunciation. Notably, not all features and dialogs could be configured independently, and Aaron GmbH still had to be contacted for non-standardized requests.

While the functionality was extended based on the study results, the main evaluation reported below was conducted based on the June 2021 version of the CovBot.

### Main study

#### Recruitment of local health authorities and baseline characteristics

**Figure 1.**
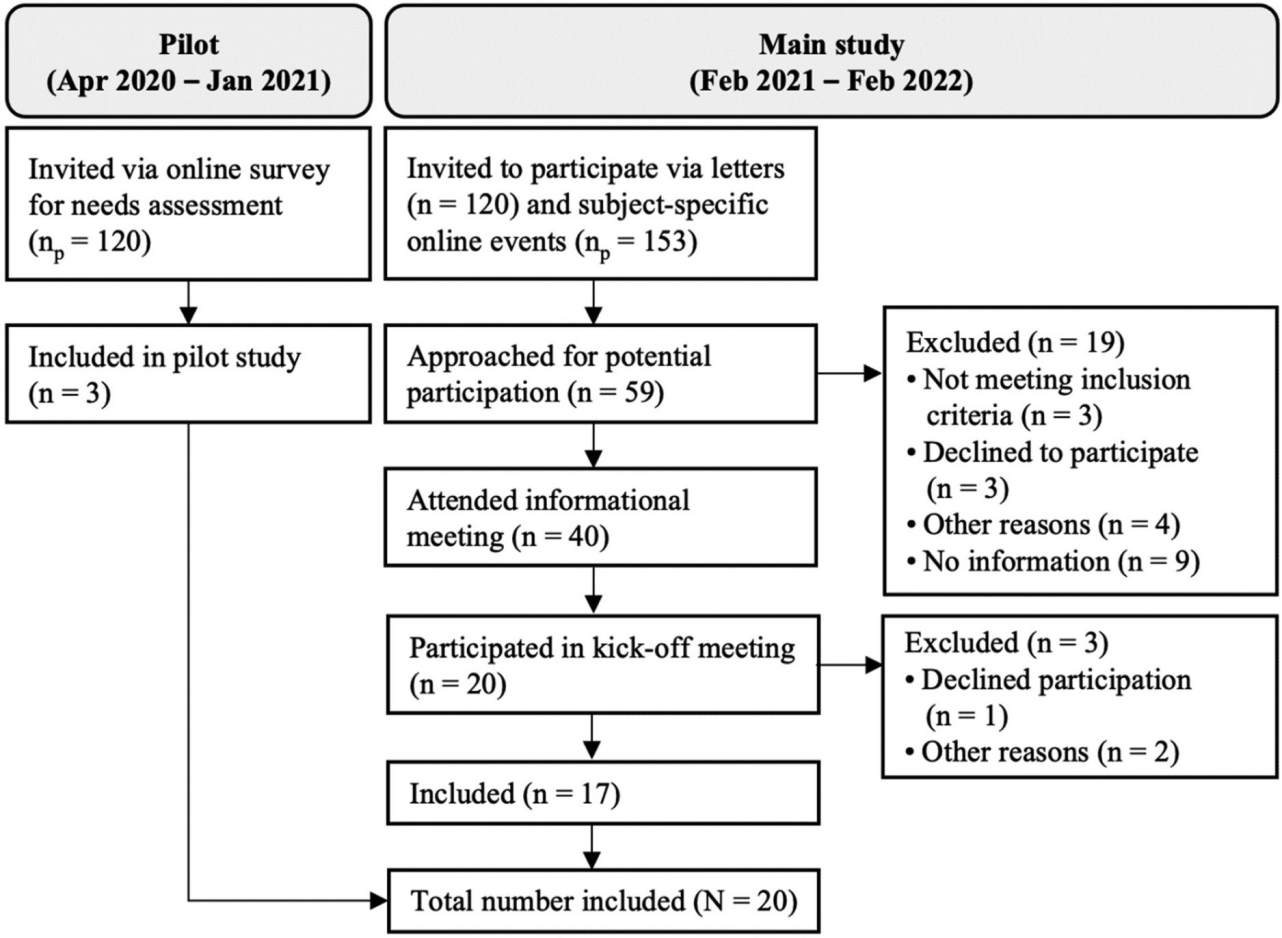
Flow chart displaying recruitment of local health authorities. *n*: number of local health authorities; n_p_: number of participants from local health authorities.

We had personal contact with 59 local health authorities for potential participation. Of those, 40 attended initial informational meetings for participation and ultimately 20 were successfully included in the study, serving a combined 6.1 million inhabitants of Germany as per the sum of number of citizens in each area of responsibility (see Figure 1).

**Figure 2.**
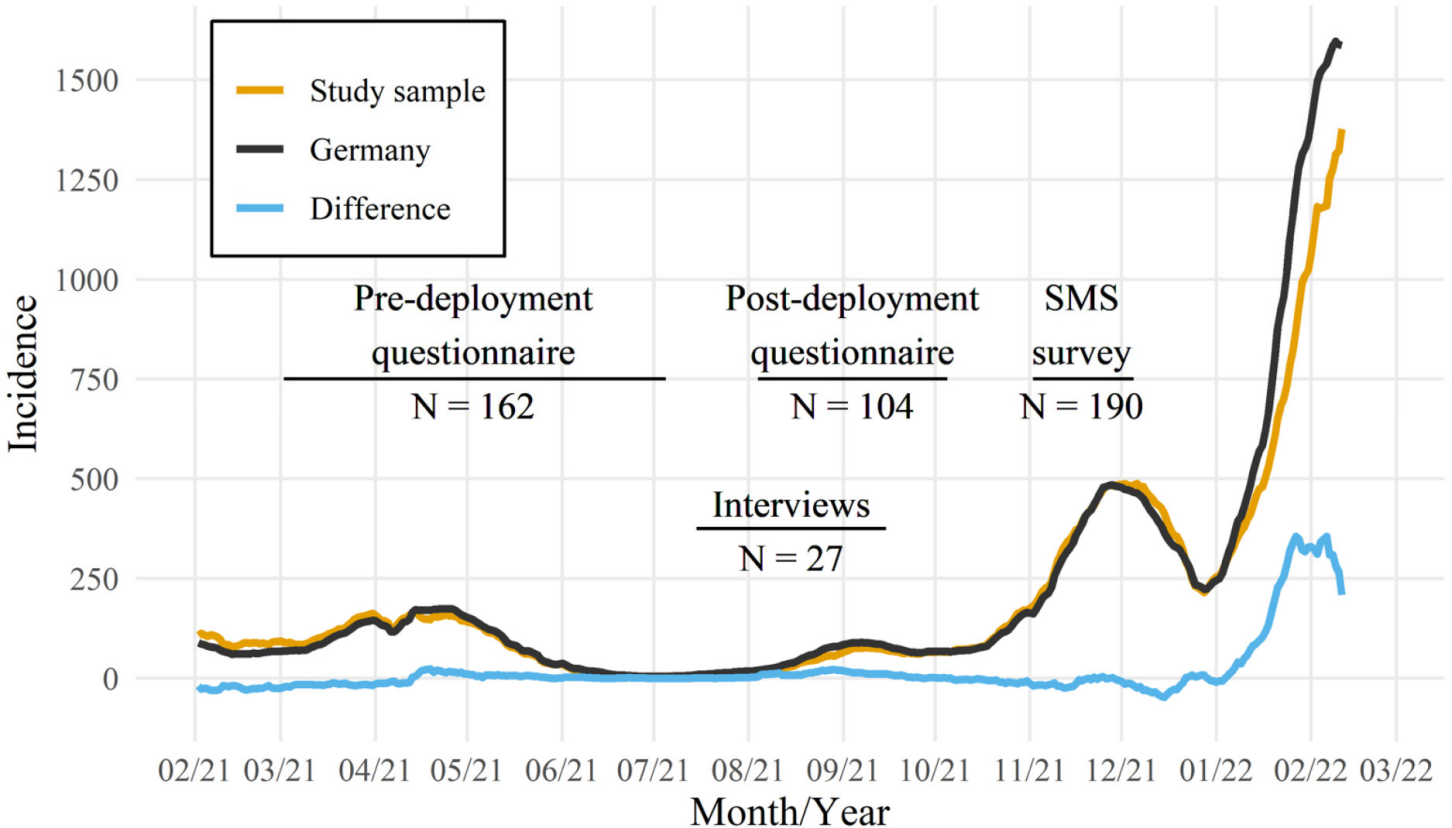
COVID-19 incidence in Germany and the study sample as reported by the federal Robert Koch Institute and data collection over the study period. Incidences are reported per seven days per 100,000 inhabitants.

The distribution of local health authorities was largely balanced in terms of size and region across Germany (see Table 1), resulting in a representative study sample of local health authorities with similar incidences of COVID-19 in 2021 compared to the whole country (see Figure 2). Predominantly local health authorities from rural areas, which are part of a district administration (in Germany, these are the primary administrative subdivisions which include multiple municipalities but are not part of a city), were included. One health authority discontinued the use of the CovBot in November 2021 due to technical problems which resulted from a sharp increase in call volume during that period (see Figure 2). However, they remained in the study for further surveys and interviews. No local health authority stopped study participation.

**Table 1.** Characteristics of participating local health authorities.

Category	Local health authorities (*N* = 20)
**Region, *n***	
North | east | south | west	4 | 5 | 7 | 4
**Size^a^, *n***	
Small | medium | large	6 | 7 | 7
Inhabitants (mean)	305.510
**District administration, *n***	
Yes | no	15 | 5
**Infrastructure, *n***	
Urban | rural	7 | 13
CovBot usage discontinuation, n	1
Study dropouts, *n*	0

^a^ Defined in each case on the basis of the number of inhabitants in the area of responsibility: small: < 130,000 inhabitants; medium: ≥ 130,000 to < 350,000 inhabitants; large: ≥ 350,000 inhabitants.

*N*: total number of local health authorities; *n*: number of local health authorities.

**Table 2.** Selected sample of the most notable SMS survey results (total number of completed questionnaires was *N*  =  190).

	Respondents, *n* (%)			
	(Strongly) agree	Neither agree nor disagree	(Strongly) disagree	Not applicable or no response^b^
**Question^a^**				
My concern was resolved through the dialog with the voicebot.	49 (26)	19 (10)	81 (42)	41 (22)
My concern was not recognized.	87 (46)	15 (8)	42 (22)	46 (24)
I intend to follow the recommendations of the voicebot.	79 (42)	32 (17)	42 (22)	37 (19)
It was easy to talk to the voicebot.	113 (60)	19 (10)	51 (27)	7 (3)
I did not know how to explain my concern to the voicebot.	68 (36)	19 (10)	58 (31)	45 (23)
The voicebot cannot replace an employee.	149 (79)	13 (7)	17 (9)	11 (5)

^a^ translated from German; ^b^per question there was a maximum of three missing responses.

*n*: number of participants with this response.

#### Duration of usage

The majority of local health authorities used the CovBot productively in their hotline system from June 2021 to February 2022. The median duration of usage was 33 weeks (range: 20–53 weeks). One local health authority temporarily switched off the CovBot on 05 July 2021 due to a manageable workload during the further course of the study, and only switched it back on temporarily during periods with a larger call volume. Study participation was free of charge for the health offices as the operating costs of the CovBot were covered by a grant provided by the German Federal Ministry of Health.

#### Call volumes

A total of 1,184,462 calls were received during the study period. The median number of calls per local health authority was 44,285, ranging from 8734 to 139,530. The number of calls was highest in November 2021, with a total of 284,000 calls and 51,611 calls to a single local health authority. The lowest call volume, with 41,752 calls and only 282 calls to a single local health authority, occurred in July 2021.

### Mixed-methods evaluations

#### Timing of assessments and return rates

Out of 40 requested interviews with 20 local health authorities, 27 telephone interviews were conducted with 19 local health authorities from July to September 2021. In total, 34 employees were interviewed, 20 of whom held management positions (head of local health authority, department management, team management in IT or hotline service) and 14 employees without management positions in IT (*n*  =  3) or hotline service (*n*  =  11). A total of 162 employees in the hotline service from 18 local health authorities answered the questionnaire predeployment, mainly in June 2021, and 104 employees in the hotline service from 20 local health authorities answered the questionnaire postdeployment mainly in early August 2021. Mean response rates of pre- and postdeployment surveys were 67% and 53%, respectively. However, due to constant changes in staff organization and the anonymous nature of the questionnaires, it remains unclear how much overlap there is for participating employees across the two survey dates.

Thirteen local health authorities participated in the SMS survey for callers. From 2 November to 5 December 2021, the CovBot invited 2388 callers to participate, of whom 940 consented to receive an SMS with the survey link which resulted in 190 completely answered surveys (response rate: 8%). Almost all project participants were involved in the needs analysis as part of the regular unstructured feedback meetings and prioritization of improvement proposals was made by all 20 participants in management positions.

#### Implementation

The median time from the initial informational interview to end-to-end deployment was eight weeks, varying greatly between local health authorities (range: 3–21 weeks). The median time period of implementation was 12 weeks (range: 3–21 weeks) before establishing a self-service configuration and five weeks (range: 3–10 weeks) after establishing a self-service configuration. The majority of local health authorities with above-average implementation time did not mention any unusual technical or organizational challenges. However, a few health authorities reported a delay due to compatibility problems with the telephone provider at the beginning of the study, which could be resolved by a change of provider organized by Aaron GmbH. For individual local health authorities, delays were attributed to lengthy internal decision-making processes. The information configured to be provided by the CovBot varied widely, including topics like general information about COVID-19, testing information, positive test result, and vaccination.

#### Qualitative evaluations of CovBot performance

##### Interviews with staff members

The majority of interviewed staff felt that the CovBot had relieved the hotline service during the COVID-19 pandemic in the summer of 2021. Some of them explicitly based this perception on the proportion of calls forwarded to an employee in relation to all incoming calls, which Aaron GmbH used as an indicator of call reduction during the regular feedback meetings. Moreover, the enhanced availability on weekends and outside opening hours was pointed out by some local health authorities. Technical issues were rarely raised in the interviews, but it was very critical for the local health authorities during the pandemic when they did occur. Some local health authorities were concerned that updating the FAQ texts for the CovBot was occasionally very time-consuming, especially when regulations changed steadily. The participants also mentioned that the public was better informed about COVID-19 issues at the time of the July to September 2021 interviews compared to the earlier phases of the pandemic. This meant that caller requests had become more complex and often required individual responses or human empathy, in contrast to the simpler and more redundant nature of questions, which arose toward the beginning of the pandemic. The interviewees valued the self-service configuration for enabling the constant adaptation of the FAQs to the rapidly changing local regulations, guidelines, and recommendations. Throughout the interviews, it was evident that the caller's satisfaction had a major impact on the acceptance of the CovBot by the local health authorities. Independent of the question of whether the CovBot reduced the calls requiring an employee response, interviewees sporadically described the conversation atmosphere as more pleasant after a preceding dialog with the CovBot.

**Caller's free-text narratives** In the SMS survey among callers in November 2021 with 190 fully completed questionnaires, there was a free-text narrative field asking “The communication with the voicebot would have been easier if…,” for which 63 participants provided a response. They wrote, for example, that contact with a staff member was desired for more individual and complex concerns. Some callers also criticized the inaccuracy of intent recognition and situations where no forwarding to an employee was possible. Moreover, some callers mentioned poor call quality and dropped calls.

#### Quantitative evaluations of CovBot performanceStaff member surveys

**Figure 3.**
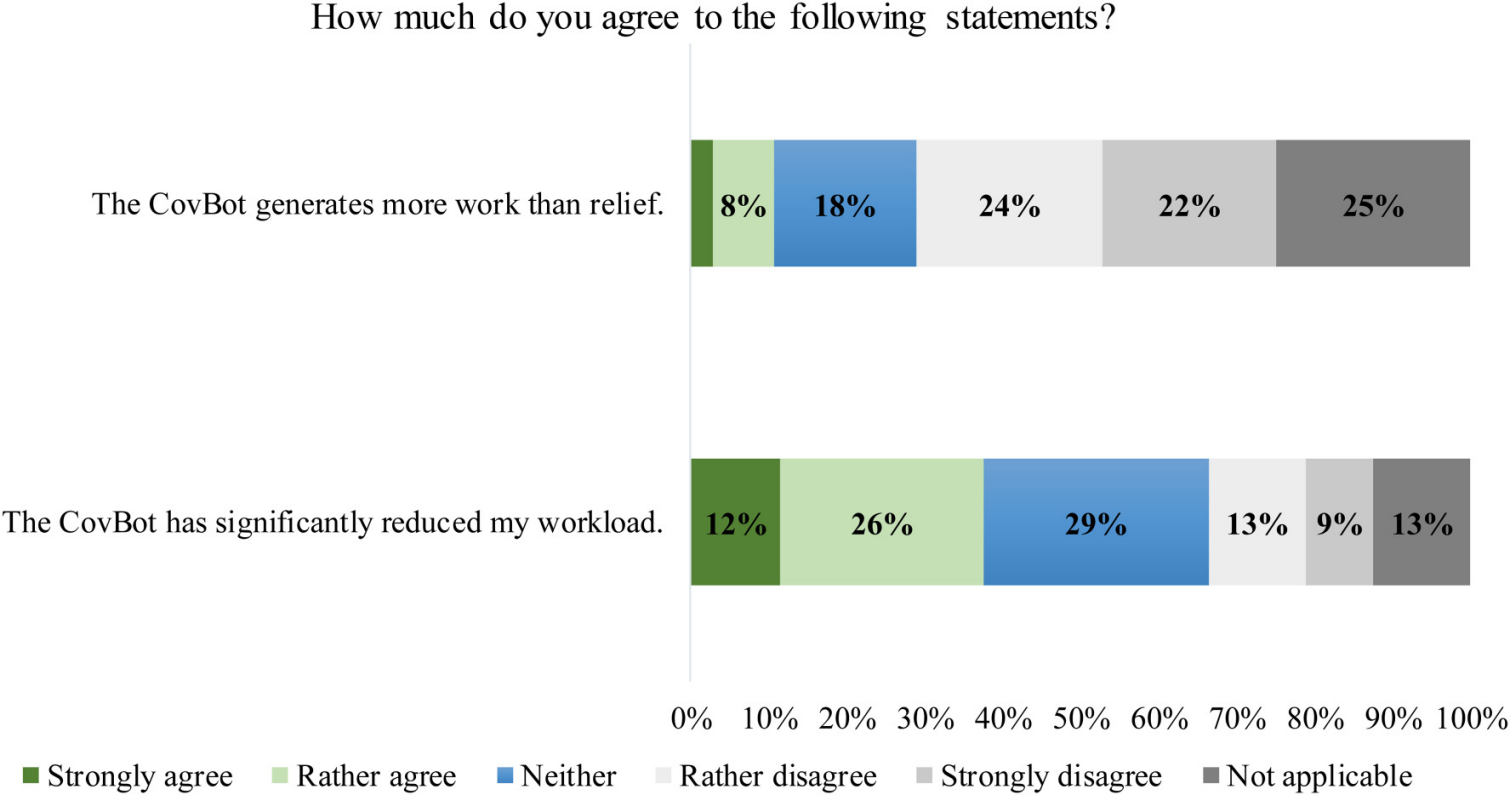
Staff member survey responses regarding an explicit reduction in workload through the CovBot. Questions were translated from German. Based on *N*  =  104 completed questionnaires.

According to the postdeployment questionnaire among telephone service staff, the CovBot did not generate more work than relief (11% [11/104] agreement). The statement that the staff's workload was noticeably reduced received more agreement (38% [39/104]) than disagreement (21% [22/104]) (see Figure 3). On the other hand, when comparing pre- and postdeployment questionnaires on the item of whether the CovBot was expected to or led to a noticeable reduction in workload, the proportion of those who agreed decreased by 22% (from 97/162 to 39/104). Along with this, 65% (68/104) of postdeployment respondents attributed the reduction in workload to the general decrease in the incidences of COVID-19 rather than to the CovBot.

#### Caller's survey responses

Regarding intent recognition, while 49 (26%) surveyed callers indicated that their question was answered adequately, there was also a high percentage of unrecognized concerns (46% [87/190]). Thereby, only 23 (12%) self-reported that they had an accent or dialect, and 15 (8%) expressed a desire for using a language other than German. The majority had mentioned their concern in only one word at least once (125 [66%]). A total of 79 (42%) agreed to the statement that they would follow the voicebot's recommendations. While 113 (60%) respondents agreed that it was easy to talk to the voicebot, 68 (36%) said they did not know how to explain their concerns. Nevertheless, about half (100 [52%]) agreed that they were satisfied with the dialog guidance. A total of 149 (79%) respondents felt that the voicebot could not replace an employee (see Table 2).

#### Anonymized metadata of the CovBot

Based on the anonymized metadata of 1,184,462 processed calls across all included local health authorities over the study period, 15% were hung up immediately (min: 3%; max: 34%), in 55% an intent was recognized and in 48% an FAQ answer was provided. Of all calls, 32% were hung up after listening to such an FAQ answer. The median proportion of calls wanting to be forwarded across all local health authorities was 51% (min: 7%; max: 78%; 61% without directly hung up calls); 63% of these forwarding attempts were successful and 37% failed. The metadata showed a median of 31% unrecognized concerns across all included local health authorities (min: 15%; max: 50%).

#### Test calls

Of the 300 conducted test calls, 191 assessed predefined intents and 109 unknown (i.e. not predefined) intents. For the predefined intents, 152 (80%) were correctly recognized after the initial caller utterance. Upon modified repetition of the utterance, this number increased to 166 (87%). Correct classifications increased further to 176 (92%) considering acceptable misclassifications as judged by the research team (e.g. if the test caller said “I had contact with a person who tested positive for covid” to reach the predefined intent “COVID-19 contact,” but the CovBot recognizing “general information on COVID-19”). Regarding the robustness to unexpected inputs, in 32 of the 109 calls (29%) a predefined intent was recognized despite these prompts not relating to any predefined intent.

#### Further development of the CovBot identified by needs analysis

**Figure 4.**
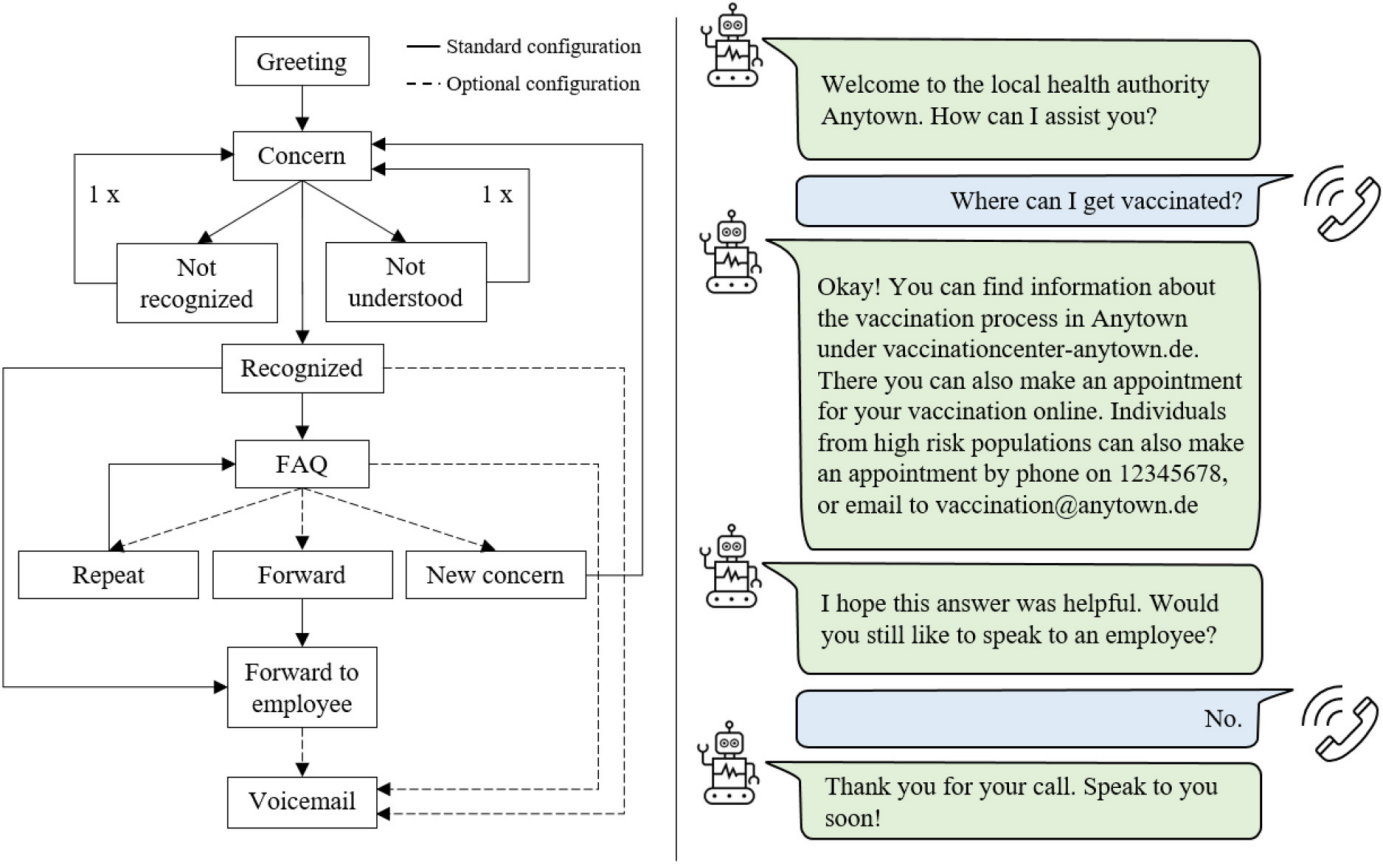
Final Q&A dialog process and exemplary conversation.

The needs analysis in July 2021 revealed a total of 16 suggestions for the further development of the CovBot. In the subsequent prioritization, the following features received the most votes: (a) a larger set of predefined concerns, (b) two intents within one call, (c) independent editing of keywords for intents, (d) enhancement of the statistics function, and (e) an answer repetition function. The company Aaron GmbH subsequently implemented these features and provided the local health authorities with an updated version of the CovBot in January 2022 (for a schematic illustration of the final Q&A process, see Figure 4). Most of the adaptations made were related to the performance. Due to the limited study period, the adapted CovBot was not reevaluated.

Finally, the needs analysis revealed a number of topics with a lower priority which were not taken into consideration due to limited time and financial constraints: more reliable intent recognition, intent recognition in voice messages, multilingualism, audio playback of recorded voice messages, and rights management for the WebApp.

## Discussion

### Principal results

The goal of this study was to evaluate the use of a voicebot by local health authorities in Germany during the COVID-19 pandemic, particularly focusing on whether a perceptible relief of staff in the hotline service could be achieved. The assessed voicebot, the CovBot, was developed in an emergency effort during the COVID-19 pandemic mainly to support answering FAQs and, if necessary, forwarding callers to an available employee. Twenty local health authorities from all over Germany participated in the study, actively using the voicebot in their hotline service over a period of 5 to 12 months. The CovBot processed almost 1.2 million calls during the study period. To provide a holistic evaluation including the user perspective and acceptance, we applied a mixed-methods approach with personal telephone interviews and online surveys among the local health authority staff and callers. Additionally, we analyzed anonymized metadata of the CovBot. Our results contribute new aspects to the existing evidence on automated telephone communication in the healthcare sector.^8–10,28–30^

The active participation in the study and contribution of suggestions for further development of the CovBot by employees of the local health authorities in Germany showed an overall positive attitude toward a voicebot that was designed according to their actual needs and requirements. Across all surveys, it became evident that fulfilling their population-relevant mandate to contain the pandemic via local oversight of infection hygiene measures and maintaining caller satisfaction were top priorities.

The interviews and surveys suggested that the local health authorities hardly received any complaints about the voicebot from callers as long as there were no technical problems (e.g. with intelligibility due to poor call quality). Technical problems occurred as a result of telephone line overload, particularly during the rapid increase in the incidences of COVID-19 in November 2021. These were resolved by enhancing the line capacities. However, the SMS survey conducted among callers during this period reflected these technical problems and yielded a mixed picture with an indication that the voicebot cannot replace an employee, especially for more complex and individual concerns. Similar findings on the acceptability of a chatbot were obtained in studies with citizens in the United Kingdom, concluding that the use of chatbots should not be recommended for more severe conditions like coughing up blood or severe depression,^31^ and discussing it as a supplementary service rather than a replacement.^32^ In addition, the interviewed local health authority staff confirmed that concerns which could not be answered by the voicebot were often the result of overcomplex as well as individualized questions or came from callers who needed human empathy. The hotline staff suggested that the complexity and individuality of concerns had presumably increased since the beginning of the pandemic due to a better understanding of the disease. A similar hypothesis was formulated based on data from conversational agents deployed during the COVID-19 pandemic in nine countries via the platform “Watson Assistant” (IBM Watson Health).^8^ The possibly less complex concerns during the beginning of the pandemic could also explain the more promising results of our pilot study reflecting a slightly greater relief of hotline service staff in 2020, whereby only three local health authorities were involved in this pilot. A particular potential for a voicebot could therefore exist at the beginning of acute crisis situations when the call volume rapidly exceeds the capacity of telephone hotlines, while question-and-answer dialogs remain relatively stable and predictable.

Our study showed the importance of an available blueprint allowing a voicebot to be activated quickly with the least possible need for additional resources from local health authorities for acute situations. The ability to standardize and automate the implementation process is an important prerequisite that we pursued during the project. The self-service configuration added in June 2021 included a default set of configurations for the initial deployment and led to some degree of standardization and automation. The median implementation duration was subsequently reduced by approximately seven weeks, although there was great variation between individual local health authorities independent of the self-service configuration. One reason might be that some local health authorities changed the predefined FAQ texts before the initial activation of the CovBot. Another important aspect to be considered is that Germany's regulations, guidelines, and recommendations differ by state. To account for this variability, local health authorities from all over Germany were recruited in order to incorporate the differences between federal states as well as other unpredictable external factors. However, the actual influence of the self-service configuration on the voicebot implementation time could not be precisely quantified. Given the frequent demand for FAQ text adaptations by health authority staff, we found the self-service configuration to be one of the most important factors to ensure smooth and continuous usability. Similar experiences concerning the need for adaptations of information during the pandemic have been reported by the authors of “Chloe,” a chatbot for questions concerning COVID-19 in Canada.^28^ The project described the increasingly broad and rapidly changing information needs as one of the biggest challenges in a complex and multijurisdictional landscape which is similar to that of Germany. However, if individual local health authorities need to adapt the FAQ texts manually, as is the case with the CovBot, the peaks in workload due to high call volumes will likely coincide with the need for adjustments, thereby limiting the performance in terms of reduced workload.

Our results indicate that the CovBot version from June 2021 may have contributed to a relief of the hotline service at local health authorities in Germany during the COVID-19 pandemic. This overall assessment was mainly based on the interviews and surveys among the local health authorities’ staff, which were (at least partly) influenced by statistics presented by Aaron GmbH during regular feedback meetings. Interpreting the proportion of calls forwarded to an employee in relation to all calls as an indicator for an overall reduction in calls is not a sufficient indicator to estimate the system's performance. With the data available within the study, it was not possible to validly quantify the proportion of calls that were conclusively answered by the CovBot. The analyses of the caller survey showed a mixed picture, which was partly due to the technical problems of the CovBot that occurred during the time of the survey. The analyses of anonymized metadata of the CovBot yielded similar, although slightly more positive results. For example, the metadata showed a slightly lower proportion of 31% unrecognized intents across local health authorities compared to the caller survey with 46% of positive respondents. Interestingly, one-third of calls were hung up after listening to an FAQ. This could be an indicator of satisfactorily answered concerns, however, it was slightly higher than the 26% positive answers found in the SMS survey. The 51% proportion of calls forwarded to an employee across all local health authorities reflects the importance attributed to direct contact with a staff member, as 79% of callers replied that a health authority employee was irreplaceable. At the same time, 37% of forwarding attempts failed and couldn't be picked up by the health offices. Due to technical differences in telephone systems as well as high fluctuations in call volume, staff capacity, and opening hours throughout the study period, this finding is difficult to interpret and needs further investigation.

Noticeably, the participating local health authorities did not define intents for all possible concerns, but chose the most common queries since the maintenance of predefined intents was time-consuming. This is consistent with the result of our test calls. Taking into account acceptable misclassification in a sensitivity analysis, the proportion of predefined intents that were recognized ranged from 80% to 92%. Nevertheless, besides the high effort associated with the manual modification of increasingly complex FAQ texts, our broad evaluation suggests that the accuracy of the intent recognition has limited the performance of the CovBot. Both the accuracy of intent recognition and the ability for more complex interactive dialogs could be improved substantially by continuous analysis of callers’ intents (and thus, at least short-term storage of dialog content) via a self-learning AI system.^33,34^ With the remarkable pace of developments in AI and the recent appearance of large language models (like ChatGPT^35^), we would expect a much better intent recognition and the ability to handle more complex concerns nowadays. The availability of these models could improve the intent recognition of even more complex concerns. Manual development of predefined FAQ answers would most probably be less prevalent, as these models could be expanded using local health authorities’ existing written information (e.g. on a webpage or in guidelines). Automatic translation of responses into multiple languages and easy-to-comprehend language are novel additions to those models, possibly further increasing their accessibility. Given that AI is poised to take over more tasks, the responsibility to investigate associated risks increases. We should be cautious not to rush the implementation of every available functionality before we fully understand their adverse implications. This particularly holds for the domain of public health.

It became apparent that for a holistic performance assessment it is essential to bring together the different results of the qualitative and quantitative research components. For example, the employees’ perception of a reduction in workload was not exclusively dependent on call volume metrics, but also on the atmosphere and quality of the conversation. Since some of the interviewees were also responsible for the internal implementation of the CovBot, a certain bias due to the expectations of the respondents cannot be ruled out.^36,37^ However, the hotline service staff not otherwise involved in the study procedures perceived a trend toward reduced workload due to the CovBot. The mixed-methods approach further allowed us to contribute to the current implementation science theories by describing which contextual factors in our study were most decisive for a successful deployment of the CovBot.^12^ Our strong emphasis on user-centeredness and analysis of user behavior revealed insights into parameters relevant to technology acceptance including underlying theories^13,32,38^ for recipients (i.e. callers) as well as adopters (i.e. health authority staff). The results of our study are in line with a systematic review of Kelly et al. (2023)^14^ who found that the perceived usefulness, performance expectancy, attitudes, trust, and effort expectancy were positive predictors of the satisfactory uptake of AI across multiple industries.

### Limitations

Several limitations deserve mentioning. Given the emergency to support overwhelmed local health authority hotlines, we performed what should be considered an exploratory noncontrolled study and did not prospectively apply a theory-driven implementation approach.^39^ Our findings mainly provide a broad description of employees’ as well as callers’ user experiences and acceptance of the use of a voicebot in local health authorities in Germany investigating whether relief of workload for the telephone hotline staff could be achieved. Due to the limited time, we were only able to evaluate an intermediate version of the CovBot and not a more advanced later version, nor could we compare it to other voicebots with similar functionality. We were also limited by a cautious reluctance toward functionalities with a need for extensive data protection reviews. Furthermore, since the surveys were anonymous and the staff structure differed between the two time points of assessment, we could not conduct a meaningful before and after comparison. The response rate for the SMS survey was only 8%, probably including a relevant nonresponse bias.^40,41^ It should be noted that the interviews and surveys were conducted at a time when incidences of COVID-19 were comparatively low in Germany. As these results cannot be extrapolated to other phases of the pandemic, it remains unclear to what extent the decrease in case numbers contributed to the reported relief.

### Conclusion

This study assessed a voicebot whose primary purpose was to answer FAQs to provide supplementary support to the hotline service of local health authorities in Germany during the COVID-19 pandemic. While our results outline its potential to relieve hotline staff, a personal conversation of those seeking assistance with health authority staff members cannot be fully replaced, especially due to often complex concerns and the need for human empathy. Consequently, a forwarding option to an employee and sufficient availability of staff is essential to address the information needs of the public. An ability to answer more complex intents using a voicebot would be technically possible but could not be realized due to high data protection requirements. Their implementation would have taken too much time in the emergency situation during the early course of the COVID-19 pandemic. The user perspective and acceptance among the local health authorities’ staff and callers showed that the perceived relief for the hotline service not only depended on call volume metrics, but also on soft indicators such as the atmosphere and quality of the conversation. In the broader scope of the ongoing digitalization of healthcare systems, it remains to be seen which other tasks such intelligent conversational agents could perform in comparable crises and regular care beyond the COVID-19 pandemic. Given the confidentiality of health data, such tools should only be used after a thorough evaluation of their benefits, risks, costs, and acceptance.

## Supplemental Material

sj-docx-1-dhj-10.1177_20552076231180677 - Supplemental material for Prospective mixed-methods study evaluating the potential of a voicebot (CovBot) to relieve German health authorities during the COVID-19 infodemic Click here for additional data file. Supplemental material, sj-docx-1-dhj-10.1177_20552076231180677 for Prospective mixed-methods study evaluating the potential of a voicebot (CovBot) to relieve German health authorities during the COVID-19 infodemic by Vanessa Voelskow, Claudia Meßner, Tobias Kurth, Amelie Busam, Toivo Glatz and Natalie Ebert in DIGITAL HEALTH

sj-docx-2-dhj-10.1177_20552076231180677 - Supplemental material for Prospective mixed-methods study evaluating the potential of a voicebot (CovBot) to relieve German health authorities during the COVID-19 infodemic Click here for additional data file. Supplemental material, sj-docx-2-dhj-10.1177_20552076231180677 for Prospective mixed-methods study evaluating the potential of a voicebot (CovBot) to relieve German health authorities during the COVID-19 infodemic by Vanessa Voelskow, Claudia Meßner, Tobias Kurth, Amelie Busam, Toivo Glatz and Natalie Ebert in DIGITAL HEALTH

sj-docx-3-dhj-10.1177_20552076231180677 - Supplemental material for Prospective mixed-methods study evaluating the potential of a voicebot (CovBot) to relieve German health authorities during the COVID-19 infodemic Click here for additional data file. Supplemental material, sj-docx-3-dhj-10.1177_20552076231180677 for Prospective mixed-methods study evaluating the potential of a voicebot (CovBot) to relieve German health authorities during the COVID-19 infodemic by Vanessa Voelskow, Claudia Meßner, Tobias Kurth, Amelie Busam, Toivo Glatz and Natalie Ebert in DIGITAL HEALTH

sj-docx-4-dhj-10.1177_20552076231180677 - Supplemental material for Prospective mixed-methods study evaluating the potential of a voicebot (CovBot) to relieve German health authorities during the COVID-19 infodemic Click here for additional data file. Supplemental material, sj-docx-4-dhj-10.1177_20552076231180677 for Prospective mixed-methods study evaluating the potential of a voicebot (CovBot) to relieve German health authorities during the COVID-19 infodemic by Vanessa Voelskow, Claudia Meßner, Tobias Kurth, Amelie Busam, Toivo Glatz and Natalie Ebert in DIGITAL HEALTH

sj-docx-5-dhj-10.1177_20552076231180677 - Supplemental material for Prospective mixed-methods study evaluating the potential of a voicebot (CovBot) to relieve German health authorities during the COVID-19 infodemic Click here for additional data file. Supplemental material, sj-docx-5-dhj-10.1177_20552076231180677 for Prospective mixed-methods study evaluating the potential of a voicebot (CovBot) to relieve German health authorities during the COVID-19 infodemic by Vanessa Voelskow, Claudia Meßner, Tobias Kurth, Amelie Busam, Toivo Glatz and Natalie Ebert in DIGITAL HEALTH

sj-docx-6-dhj-10.1177_20552076231180677 - Supplemental material for Prospective mixed-methods study evaluating the potential of a voicebot (CovBot) to relieve German health authorities during the COVID-19 infodemic Click here for additional data file. Supplemental material, sj-docx-6-dhj-10.1177_20552076231180677 for Prospective mixed-methods study evaluating the potential of a voicebot (CovBot) to relieve German health authorities during the COVID-19 infodemic by Vanessa Voelskow, Claudia Meßner, Tobias Kurth, Amelie Busam, Toivo Glatz and Natalie Ebert in DIGITAL HEALTH

sj-docx-7-dhj-10.1177_20552076231180677 - Supplemental material for Prospective mixed-methods study evaluating the potential of a voicebot (CovBot) to relieve German health authorities during the COVID-19 infodemic Click here for additional data file. Supplemental material, sj-docx-7-dhj-10.1177_20552076231180677 for Prospective mixed-methods study evaluating the potential of a voicebot (CovBot) to relieve German health authorities during the COVID-19 infodemic by Vanessa Voelskow, Claudia Meßner, Tobias Kurth, Amelie Busam, Toivo Glatz and Natalie Ebert in DIGITAL HEALTH
