# Supplementary material for: Prospective mixed-methods study evaluating the potential of a voicebot (CovBot) to relieve German health authorities during the COVID-19 infodemic
Source: Digit Health. 2023 Jun 7;9:20552076231180677. doi: 10.1177/20552076231180677 (PMC10262654; doi:10.1177/20552076231180677)
Supplement: sj-docx-1-dhj-10.1177_20552076231180677 - Supplemental material for Prospective mixed-methods study evaluating the potential of a voicebot (CovBot) to relieve German health authorities during the COVID-19 infodemic [file sj-docx-1-dhj-10.1177_20552076231180677.docx]

**Willkommen bei der Umfrage zum COVID-19-Hotline-Assistenten für Gesundheitsämter (CovBot) in Deutschland. Die vorliegenden Fragen bezieht sich auf die letzte komplette Arbeitswoche, bevor der CovBot in Ihrem Gesundheitsamt in Betrieb genommen wurde. Der Fragebogen sollte nicht mehr als 2 bis 4 Minuten Ihrer Zeit in Anspruch nehmen. Mit Ihrer Teilnahme tragen Sie dazu bei, wirkungsvolle Maßnahmen zur Entlastung der Telefonhotlines deutscher Gesundheitsämter zu identifizieren.**

**Wir danken Ihnen im Voraus für Ihre Beteiligung!**

**Ich habe die oben stehenden Informationen gelesen. Mir ist bewusst, dass meine Teilnahme freiwillig ist und dass ich sie jederzeit ohne Angabe von Gründen und ohne nachteilige Folgen für mich abbrechen kann. Meine Antworten werden anonym ausgewertet, dabei können keine Rückschlüsse auf meine Person gezogen werden.**

**Mit Klick auf "Weiter" stimme ich der Nutzung meiner Daten für die oben genannten Zwecke zu und willige zur Teilnahme ein.**

# A1. Besteht Ihre Hauptaufgabe im Gesundheitsamt darin, die Telefonhotline zu betreuen?

Ja

# B1. Wurden Sie extra für die Betreuung der Telefonhotline eingestellt?

Nein

Ja

# C1. Da Ihre Hauptaufgabe nicht die Betreuung der Telefonhotline ist, bitte schätzen Sie, ...

Nein

wie viele Stunden Sie in Ihrer letzten kompletten Arbeitswoche COVID-19-bedingt außerhalb Ihres eigentlichen Tätigkeitsbereichs gearbeitet haben.

6-10h 11-15h 16-20h >20h

Keine Angabe

# D1. Wenn Sie an Ihre letzte komplette Arbeitswoche und die Anrufe denken, die Sie persönlich entgegengenommen haben, wie hoch schätzen Sie, ...

…war der Anteil der Anrufe mit Anliegen, für die Ihr Gesundheitsamt eigentlich nicht zuständig ist?

…war der Anteil der Anrufe mit allgemeinen Fragen, die in ähnlicher Form häufig gestellt werden und/oder auf Ihrer Website bereits beantwortet werden (sog.

FAQ/Häufig gestellte Fragen)?

0-19% 20-39% 40-59% 60-79% 80-100%

Nicht zutreffend

...war der Anteil der Anrufe, bei denen eine Einstufung (Triage) anhand von Symptomen, Kontakt zu Risikopersonen/-regionen und sonstigen Kriterien erforderlich war, z.B. nach RKI-Richtlinien?

...war der Anteil der Anrufe, bei denen es um die Impfung gegen COVID-19 ging (Termin, Informationen,

Bedenken etc.)?

...war der Anteil Ihrer Arbeitszeit, den Sie mit COVID-19 spezifischen Anfragen in der Telefonhotline

verbracht haben?

...war der Anteil Ihrer Arbeitszeit, den Sie mit der Erstellung und Pflege der Dokumentation von Anrufen

rund um COVID-19 verbracht haben?

0-19% 20-39% 40-59% 60-79% 80-100%

Nicht zutreffend

# E1. Wie sehr stimmen Sie den folgenden Aussagen zu?

Stimme

Stimme

Es gibt keine Barrieren für Bürger*innen, unser Gesundheitsamt telefonisch zu erreichen.

Seit Ausbruch der COVID-19 Pandemie ist es üblich

geworden, Überstunden zu machen.

Ich empfand den Telefondienst in meiner letzten kompletten Arbeitswoche als sehr belastend.

Seit Ausbruch der COVID-19 Pandemie ist es üblich geworden, in anderen Arbeitsbereichen eingesetzt zu

werden.

In der letzten Woche ist viel Arbeit unerledigt liegen

geblieben.

Der CovBot kann eine relevante Lösung zur Entlastung

unseres Telefondienstes sein.

Ich akzeptiere und unterstütze die Einführung eines Sprachbots zur Entlastung der Telefonhotline in meinem

Gesundheitsamt.

Ich erwarte, dass der CovBot meine Arbeitsbelastung

deutlich reduziert.

Stimme voll zu

Stimme eher zu

Weder noch

eher nicht zu

gar nicht zu

Nicht zutreffend

# F1. Da Sie der Aussage bezüglich liegen gebliebener Arbeit (eher) zustimmen, schätzen Sie bitte, ...

wie viele Überstunden nötig gewesen wären, um die bei

Ihnen angefallene Arbeit zu erledigen.

3-5h 6-8h 8-11h >12h

Keine Antwort

**Vielen Dank für Ihre Teilnahme! Für Fragen und weitere Informationen stehen wir Ihnen gerne per E-Mail zur Verfügung:** [**covbot@charite.de**](mailto:covbot@charite.de)
