# Supplementary material for: Prospective mixed-methods study evaluating the potential of a voicebot (CovBot) to relieve German health authorities during the COVID-19 infodemic
Source: Digit Health. 2023 Jun 7;9:20552076231180677. doi: 10.1177/20552076231180677 (PMC10262654; doi:10.1177/20552076231180677)
Supplement: sj-docx-3-dhj-10.1177_20552076231180677 - Supplemental material for Prospective mixed-methods study evaluating the potential of a voicebot (CovBot) to relieve German health authorities during the COVID-19 infodemic [file sj-docx-3-dhj-10.1177_20552076231180677.docx]

**Willkommen bei der Umfrage zum Covid-19-Hotline-Assistenten für Gesundheitsämter (CovBot) in Deutschland. Dieser Fragebogen bezieht sich auf Ihre letzte komplette Arbeitswoche. Der Zeitaufwand für diesen Fragebogen beträgt 4 bis 8 Minuten. Mit Ihrer Teilnahme tragen Sie dazu bei, wirkungsvolle Maßnahmen zur Entlastung der Hotlines deutscher Gesundheitsämter zu finden und den CovBot weiter zu entwickeln. Vielen Dank im Voraus für Ihre Beteiligung!**

**Ich habe die oben stehenden Informationen gelesen. Mir ist bewusst, dass meine Teilnahme freiwillig ist und dass ich sie jederzeit ohne Angabe von Gründen und ohne nachteilige Folgen für mich abbrechen kann. Meine Antworten werden anonym ausgewertet, dabei können keine Rückschlüsse auf meine Person gezogen werden.**

**Mit klick auf "Weiter" stimme ich der Nutzung meiner Daten für die oben genannten Zwecke zu und willige zur Teilnahme ein.**

# A1. Besteht Ihre Hauptaufgabe im Gesundheitsamt darin, die Telefonhotline zu betreuen?

Ja

# B1. Wenn Sie an Ihre letzte komplette Arbeitswoche und die Anrufe denken, die Sie persönlich entgegengenommen haben, wie hoch schätzen Sie...

Nein

… war der Anteil der Anrufe mit Anliegen, für die Ihr Gesundheitsamt eigentlich nicht zuständig ist?

… war der Anteil der Anrufe mit allgemeinen Fragen, die in ähnlicher Form häufig gestellt werden und/oder auf Ihrer Website bereits beantwortet werden (sog.

FAQ/Häufig gestellte Fragen)?

0-19% 20-39% 40-59% 60-79% 80-100%

Nicht zutreffend

... war der Anteil der Anrufe, bei denen eine Einstufung (Triage) anhand von Symptomen, Kontakt zu Risikopersonen/-regionen und sonstigen Kriterien erforderlich war, z.B. nach RKI-Richtlinien?

...war der Anteil Ihrer Arbeitszeit, den Sie mit Covid-19 spezifischen Anfragen in der Telefonhotline verbracht

haben?

...war der Anteil Ihrer Arbeitszeit, den Sie mit der Erstellung und Pflege der Dokumentation von Anrufen

rund um Covid-19 verbracht haben?

…war der Anteil Ihrer Arbeitszeit, den Sie mit

Rückrufen verbracht haben?

# C1. Wie sehr stimmen Sie den folgenden Aussagen zu?

Stimme

Stimme

Ich empfand den Telefondienst in meiner letzten kompletten Arbeitswoche als sehr belastend.

In der letzten Woche ist viel Arbeit unerledigt liegen

geblieben.

Der CovBot hat meine Arbeitsbelastung deutlich

reduziert.

Die Reduktion der Arbeitsbelastung war nach meinem Gefühl eher durch den Rückgang der Fallzahlen als durch

den CovBot bedingt.

Stimme voll zu

Stimme eher zu

Weder noch

eher nicht zu

gar nicht zu

Nicht zutreffend

Der CovBot ist für mich einfach zu benutzen.

Ich kann mir vorstellen, dass die meisten Mitarbeiter*innen den Umgang mit dem CovBot sehr

schnell lernen können.

Ich habe generell oft technische Probleme an meinem

Arbeitsplatz.

Das Zusammenspiel zwischen dem CovBot und den verschiedenen Abläufen innerhalb des Gesundheitsamts

funktioniert reibungslos.

Der CovBot erzeugt mehr Arbeit als Nutzen.

Die Barrierefreiheit des CovBots für die Anrufer*innen

sollte noch verbessert werden.

Ein Sprachbot könnte auch an anderen Stellen im Gesundheitsamt eingesetzt werden.

Ich denke, dass ein Sprachbot, wie der CovBot, auch über die Pandemie hinaus, meine tägliche Arbeit im

Telefondienst erleichtern würde.

# D1. Da Sie der Aussage bezüglich liegen gebliebener Arbeit (eher) zustimmen, schätzen Sie bitte...

wie viele Überstunden nötig gewesen wären um die bei

Ihnen angefallene Arbeit zu erledigen.

<2h 3-5h 6-8h 8-11h >12h

Keine Antwort

# E1. Welche zusätzlichen Funktionen des CovBots verwendet Ihr Gesundheitsamt? Mehrfachantwort möglich!

Webinterface

Sprachnachrichten

Statistik

# E2. Welche zusätzlichen Funktionen oder anderen Möglichkeiten würden Sie sich vom CovBot noch wünschen? Tragen Sie bitte Ihre Wunschfunktionen ein!

Wunschfunktion 1

|  |  |  |  |  |  |  |  |  |  |
| --- | --- | --- | --- | --- | --- | --- | --- | --- | --- |

Wunschfunktion 2

|  |  |  |  |  |  |  |  |  |  |
| --- | --- | --- | --- | --- | --- | --- | --- | --- | --- |

Wunschfunktion 3

|  |  |  |  |  |  |  |  |  |  |
| --- | --- | --- | --- | --- | --- | --- | --- | --- | --- |

Wunschfunktion 4

|  |  |  |  |  |  |  |  |  |  |
| --- | --- | --- | --- | --- | --- | --- | --- | --- | --- |

Wunschfunktion 5

|  |  |  |  |  |  |  |  |  |  |
| --- | --- | --- | --- | --- | --- | --- | --- | --- | --- |

# F1. Wenn Sie an das Webinterface denken, wie sehr stimmen Sie den Aussagen zu?

Ich benutze das Webinterface gerne.

Stimme voll zu

Stimme eher zu

Weder noch

Stimme eher nicht zu

Stimme gar nicht zu

Nicht zutreffend

Ich benutze das Webinterface regelmäßig.

Ich empfinde das Webinterface als zu komplex.

Die verschiedenen Funktionen sind gut in das

Webinterface integriert.

# G1. Wenn Sie an die Sprachnachrichtenfunktion denken, wie sehr stimmen Sie den Aussagen zu?

Die Sprachnachrichtenfunktion ist von Nutzen in meinem

Arbeitsalltag in der Telefonhotline.

Die Niederschriften der Sprachnachrichten sind

inhaltlich korrekt.

Ich plane zukünftig meine Rückrufe häufiger anhand der

Niederschriften zu priorisieren.

Stimme voll zu

Stimme eher zu

Weder noch

Stimme eher nicht zu

Stimme gar nicht zu

Nicht zutreffend

# H1. Wenn Sie an die Statistikfunktion denken, wie sehr stimmen Sie den Aussagen zu?

Ich verwende die Statistikfunktion in meinem Arbeitsalltag in der Telefonhotline regelmäßig.

Stimme voll zu

Stimme eher zu

Weder noch

Stimme eher nicht zu

Stimme gar nicht zu

Nicht zutreffend

Die Statistikfunktion enthält alle relevanten Daten.

Ich kann im Allgemeinen nichts mit Statistiken anfangen.

# I1. Wenn Sie an die Gespräche mit Anrufer*innen nach Weiterleitung durch den CovBot denken, wie sehr stimmen Sie den folgenden Aussagen zu?

Die Anrufer*innen geben positive Rückmeldungen zu

dem CovBot.

Stimme voll zu

Stimme eher zu

Weder noch

Stimme eher nicht zu

Stimme gar nicht zu

Nicht zutreffend

Die Anrufer*innen beschweren sich über den CovBot.

# J1. Wie sehr stimmen Sie den folgenden Aussagen zu? Wenn sich Anrufer*innen über den CovBot beschweren, dann weil…

… sie ausschließlich mit Mitarbeiter*innen sprechen

wollten.

… der CovBot das Anliegen nicht abschließend lösen

konnte.

Stimme voll zu

Stimme eher zu

Weder noch

Stimme eher nicht zu

Stimme gar nicht zu

Nicht zutreffend

… der CovBot schwer zu verstehen war.

… der CovBot keine Mehrsprachigkeit angeboten hat.

**Vielen Dank für Ihre Teilnahme! Für Fragen und weitere Informationen stehen wir Ihnen gerne per E-Mail zur Verfügung:** [**covbot@charite.de**](mailto:covbot@charite.de)
