# Supplementary material for: Prospective mixed-methods study evaluating the potential of a voicebot (CovBot) to relieve German health authorities during the COVID-19 infodemic
Source: Digit Health. 2023 Jun 7;9:20552076231180677. doi: 10.1177/20552076231180677 (PMC10262654; doi:10.1177/20552076231180677)
Supplement: sj-docx-4-dhj-10.1177_20552076231180677 - Supplemental material for Prospective mixed-methods study evaluating the potential of a voicebot (CovBot) to relieve German health authorities during the COVID-19 infodemic [file sj-docx-4-dhj-10.1177_20552076231180677.docx]

**Willkommen bei der Umfrage zur Priorisierung der am häufigsten genannten Verbesserungswünsche am CovBot! Dieser Fragebogen beinhaltet die Verbesserungswünsche am CovBot, die uns bislang von Gesundheitsämtern am häufigsten mitgeteilt worden sind. Wir möchten Sie mit dieser Umfrage bitten, die Verbesserungswünsche für Ihr Gesundheitsamt zu priorisieren. Der Zeitaufwand für diesen Fragebogen beträgt etwa 5 Minuten.**

**Mit Ihrer Teilnahme tragen Sie dazu bei, wirkungsvolle Maßnahmen zur Entlastung der Hotlines deutscher Gesundheitsämter zu finden und den CovBot weiterzuentwickeln. Vielen Dank im Voraus für Ihre Beteiligung!**

**Ich habe die oben stehenden Informationen gelesen. Mir ist bewusst, dass meine Teilnahme freiwillig ist und dass ich sie jederzeit ohne Angabe von Gründen und ohne nachteilige Folgen für mich abbrechen kann. Meine Antworten werden anonym ausgewertet, dabei können keine Rückschlüsse auf meine Person gezogen werden.**

**Mit Klick auf "Weiter" stimme ich der Nutzung meiner Daten für die oben genannten Zwecke zu und willige zur Teilnahme ein.**

**A1. Möglichkeiten zur Weiterentwicklung des CovBots**

**Im Folgenden finden Sie Verbesserungswünsche am CovBot, die uns bislang von Gesundheitsämtern am häufigsten mitgeteilt worden sind und im Rahmen der Studie noch implementiert werden könnten.**

**Wenn Sie an den CovBot und die Situation in Ihrem Gesundheitsamt denken, mit welcher Priorität würden Sie sich wünschen…**

…, dass der Sprachbot mehrere Sprachen sprechen kann?

Nicht relevant

Niedrige Priorität

Moderate Priorität

Hohe Priorität

Höchste Priorität

Nicht zutreffend

…, dass der Sprachbot Informationen auf Wunsch der

Anrufer*innen wiederholen kann?

…, dass der Sprachbot mehr als ein Anliegen von

Anrufer*innen beantworten kann?

…, dass der Sprachbot eine natürlichere Stimme

bekommt?

…, dass der Sprachbot auch selbst aufgenommene

Sprachaufnahmen abspielen kann?

…, dass der Sprachbot zuverlässiger Anliegen erkennt?

**B1. Möglichkeiten zur Weiterentwicklung der Benutzeroberfläche (Web- Applikation) des CovBots**

**Im Folgenden finden Sie Verbesserungswünsche an der Benutzeroberfläche (Web-Applikation [WebApp] des CovBots), die uns bislang von Gesundheitsämtern am häufigsten mitgeteilt worden sind und im Rahmen der Studie noch implementiert werden könnten. Wenn Sie an den CovBot und die Situation in Ihrem Gesundheitsamt denken, mit welcher Priorität würden Sie sich wünschen…**

…, dass man in der Sprachnachrichtenanzeige direkt einsehen kann, welchem Anliegen beziehungsweise Fachbereich die Nachricht zuzuordnen ist?

…, dass man sich in der Sprachnachrichtenanzeige selbst

Notizen machen kann?

…, dass man in der WebApp kleine Anleitungen beziehungsweise Hilfetexte zur Nutzung hat?

…, dass man in der WebApp die Statistiken tagesaktuell und mit grafischen Darstellungen einsehen kann?

…, dass man in der WebApp die Gesamtanzahl der Anrufe einsehen kann, die intern weitergeleitet wurden?

…, dass man in der WebApp Anliegen pausieren kann, ohne diese direkt unwiderruflich zu löschen?

…, dass man in der WebApp selbstständig Anliegen (linke Spalte) ergänzen oder löschen kann?

…, dass man in der WebApp direkt zu Beginn bereits noch mehr Anliegen (linke Spalte) zur Verfügung hat?

…, dass man in der WebApp die den Anliegen zugrunde liegenden Schlüsselwörter einsehen kann?

…, dass man in der WebApp die den Anliegen zugrunde liegenden Schlüsselwörter bearbeiten kann?

Nicht relevant

Niedrige Priorität

Moderate Priorität

Hohe Priorität

Höchste Priorität

Nicht zutreffend

**C1. Welche Funktionen würden Sie sich neben Ihren bereits priorisierten Funktionen vom CovBot beziehungswiese der Web-Applikation noch wünschen? Bitte tragen Sie Ihre Wünsche ein.**

|  |  |  |  |  |  |  |  |  |  |
| --- | --- | --- | --- | --- | --- | --- | --- | --- | --- |

Wunsch 1

Wunsch 2

|  |  |  |  |  |  |  |  |  |  |
| --- | --- | --- | --- | --- | --- | --- | --- | --- | --- |

Wunsch 3

|  |  |  |  |  |  |  |  |  |  |
| --- | --- | --- | --- | --- | --- | --- | --- | --- | --- |

**C2. Welche weiteren Herausforderungen gibt es in Ihrem Gesundheitsamt, die nach Ihrer Einschätzung durch eine Weiterentwicklung des CovBots verbessert werden könnten?**

|  |  |  |  |  |  |  |  |  |  |
| --- | --- | --- | --- | --- | --- | --- | --- | --- | --- |

Herausforderung 1

Herausforderung 2

|  |  |  |  |  |  |  |  |  |  |
| --- | --- | --- | --- | --- | --- | --- | --- | --- | --- |

Herausforderung 3

|  |  |  |  |  |  |  |  |  |  |
| --- | --- | --- | --- | --- | --- | --- | --- | --- | --- |

**Vielen Dank für Ihre Teilnahme! Für Fragen und weitere Informationen stehen wir Ihnen gerne per E-Mail zur Verfügung:** [**covbot@charite.de**](mailto:covbot@charite.de)
