# Supplementary material for: Prospective mixed-methods study evaluating the potential of a voicebot (CovBot) to relieve German health authorities during the COVID-19 infodemic
Source: Digit Health. 2023 Jun 7;9:20552076231180677. doi: 10.1177/20552076231180677 (PMC10262654; doi:10.1177/20552076231180677)
Supplement: sj-docx-5-dhj-10.1177_20552076231180677 - Supplemental material for Prospective mixed-methods study evaluating the potential of a voicebot (CovBot) to relieve German health authorities during the COVID-19 infodemic [file sj-docx-5-dhj-10.1177_20552076231180677.docx]

Version: 1.0 **CovBot: Interview form for managers** 06.07.2021

# Manuscript title: Prospective Mixed Methods Study Evaluating the Potential of a Voicebot (CovBot) to Relieve German Health Authorities During the COVID-19 Infodemic

# Einstiegsfragen

1. Welche Auswirkungen hatte die Entwicklung der Infektionszahlen der Covid-19 Pandemie in den letzten 3 Monaten auf Ihren Zuständigkeitsbereich?
2. Würden Sie uns bitte die Organisationsstruktur des Telefondienstes in Ihrem Gesundheitsamt beschreiben (beispielsweise Anzahl Rufnummern)?

# Themenkomplex 1 – Nutzerakzeptanz, Vor- und Nachteile

1. **Funktionen:** Welche Funktionen des CovBots (z.B. Sprachnachrichtenfunktion) nutzen Sie in Ihrem Gesundheitsamt aktiv für die tägliche Arbeit?
2. **Vor- und Nachteile:** Welche Stärken und Schwächen haben der CovBot und die Web-Applikation? *Optionaler Zusatz (Funktionen): Aus welchen Gründen werden manche Funktionen nicht regelmäßig genutzt (Anknüpfung an Frage 1, Themenkomplex 1)?*
3. **Nutzerakzeptanz:** Wie zufrieden sind Sie mit dem CovBot und der Web-Applikation inklusive etwaiger zusätzlicher Funktionen?

# Themenkomplex 2 – Implementierung und Prozessintegration

1. **Implementierung und Wartung:** Wie beurteilen Sie die Implementierung und Wartung des CovBots? *Optionaler Zusatz, sofern die Implementierung des CovBots überdurchschnittlich lange gedauert hat (lange Implementierung):* Welche Aspekte führten dazu, dass die Implementierung des CovBots überdurchschnittlich lange gedauert hat, und welche Rolle haben dabei datenschutzrechtliche Bedenken gespielt?
2. **Weitere Prozessintegration:** Könnten Sie sich vorstellen den Sprachbot während Pandemien für weitere Prozesse im Gesundheitsamt zu nutzen?
3. **Barrierefreiheit:** Wie schätzen Sie die Barrierefreiheit des CovBots ein?

# Themenkomplex 3 – Ausblick

1. **Bedarf:** Gibt es Bedarf, einen Sprachbot außerhalb von Pandemien für die Telefondienste an Gesundheitsämtern einzusetzen?
2. **Finanzierung:** Wie könnte die Finanzierung eines Sprachbots außerhalb von Pandemien sichergestellt werden?
3. **Abschlussfrage:** Gibt es aus Ihrer Sicht noch wichtige Aspekte, die bisher zu wenig berücksichtigt wurden?

S e i t e 1 | 1
