# Supplementary material for: Prospective mixed-methods study evaluating the potential of a voicebot (CovBot) to relieve German health authorities during the COVID-19 infodemic
Source: Digit Health. 2023 Jun 7;9:20552076231180677. doi: 10.1177/20552076231180677 (PMC10262654; doi:10.1177/20552076231180677)
Supplement: sj-docx-6-dhj-10.1177_20552076231180677 - Supplemental material for Prospective mixed-methods study evaluating the potential of a voicebot (CovBot) to relieve German health authorities during the COVID-19 infodemic [file sj-docx-6-dhj-10.1177_20552076231180677.docx]

Version: 1.0 **CovBot: Interview form for staff** 06.07.2021

**Manuscript title: Prospective Mixed Methods Study Evaluating
the Potential of a Voicebot (CovBot) to Relieve German Health
Authorities During the COVID-19 Infodemic**

# Einstiegsfrage

1. Wie lange arbeiten Sie schon in Ihrer Position und was sind Ihre Hauptaufgaben?

# Themenkomplex 1 – Nutzerakzeptanz, Vor- und Nachteile

1. **Vor- und Nachteile:** Welche Stärken und Schwächen haben der CovBot und die Web-Applikation?
2. **Nutzerakzeptanz:** Wie zufrieden sind Sie mit dem CovBot und der Web-Applikation inklusive etwaiger zusätzlicher Funktionen?
3. **Barrierefreiheit:** Wie schätzen Sie die Barrierefreiheit des CovBots ein? Gab es Rückmeldungen von Anrufer*innen, die Sie auf das Thema Barrierefreiheit bezogen haben?

# Themenkomplex 2 – Implementierung und Prozessintegration

1. **Implementierung und Wartung:** Wie beurteilen Sie die Implementierung und Wartung des CovBots?
2. **Organisatorische beziehungsweise technische Herausforderungen:** Gab es organisatorische (für Mitarbeiter*innen Telefondienst) beziehungsweise technische Aspekte (für Mitarbeiter*innen Informationstechnologie [IT]), wegen derer die Implementierung länger gedauert hat als ursprünglich geplant?
3. **Weitere Prozessintegration:** Könnten Sie sich vorstellen den Sprachbot während Pandemien für weitere Prozesse im Gesundheitsamt zu nutzen?

# Themenkomplex 3 – Ausblick

1. **Bedarf:** Gibt es Bedarf, einen Sprachbot außerhalb von Pandemien für die Telefondienste an Gesundheitsämtern einzusetzen?
2. **Abschlussfrage:** Gibt es aus Ihrer Sicht noch wichtige Aspekte, die bisher zu wenig berücksichtigt wurden?

S e i t e 1 | 1
