# Supplementary material for: Prospective mixed-methods study evaluating the potential of a voicebot (CovBot) to relieve German health authorities during the COVID-19 infodemic
Source: Digit Health. 2023 Jun 7;9:20552076231180677. doi: 10.1177/20552076231180677 (PMC10262654; doi:10.1177/20552076231180677)
Supplement: sj-docx-7-dhj-10.1177_20552076231180677 - Supplemental material for Prospective mixed-methods study evaluating the potential of a voicebot (CovBot) to relieve German health authorities during the COVID-19 infodemic [file sj-docx-7-dhj-10.1177_20552076231180677.docx]

Version: 1.0 **CovBot: Caller survey** 06.07.2021

Manuscript title: Prospective Mixed Methods Study Evaluating
the Potential of a Voicebot (CovBot) to Relieve German Health Authorities During the COVID-19 Infodemic

Frage 1: Wenn Sie an den vorangehenden Dialog mit dem Sprachbot denken, mit dem Sie beim Gesundheitsamt gesprochen haben, wir sehr stimmen Sie den folgenden Aussagen zu?

|  | **Stimme gar nicht zu** | **Stimme eher nicht zu** | **weder noch** | **Stimme eher zu** | **Stimme voll zu** | **Nicht zutreffend** |
| --- | --- | --- | --- | --- | --- | --- |
| Ich war mit der Dialogführung des Sprachbot zufrieden. |  |  |  |  |  |  |
| Es war einfach mit dem Sprachbot zu sprechen. |  |  |  |  |  |  |
| Ich würde wieder mit dem Sprachbot sprechen. |  |  |  |  |  |  |
| Der Sprachbot kann keine*n Mitarbeiter*in ersetzen. |  |  |  |  |  |  |
| Ich hätte gern ausschließlich mit einer*m Mitarbeiter*in  gesprochen. |  |  |  |  |  |  |
| Der Sprachbot führt zu kürzeren Wartezeiten. |  |  |  |  |  |  |
| Ich habe dem Sprachbot ein Schlagwort zu meinem  Anliegen genannt. |  |  |  |  |  |  |
| Ich habe dem Sprachbot mein Anliegen in einem Satz  genannt. |  |  |  |  |  |  |
| Mein Anliegen wurde nicht erkannt. |  |  |  |  |  |  |
| Mein Anliegen wurde durch den Dialog mit dem Sprachbot beantwortet. |  |  |  |  |  |  |
| Ich werde die Empfehlungen des Sprachbots befolgen. |  |  |  |  |  |  |
| Ich wusste nicht, wie ich dem Sprachbot mein Anliegen  erklären soll. |  |  |  |  |  |  |
| Die Sprechgeschwindigkeit des Sprachbots war zu  schnell. |  |  |  |  |  |  |
| Der Sprachbot war einfach zu verstehen. |  |  |  |  |  |  |
| Ich habe einen Akzent oder Dialekt. |  |  |  |  |  |  |
| Ich hätte mir gewünscht, dass der Sprachbot eine weitere  Sprache spricht. |  |  |  |  |  |  |

Frage 2: Die Kommunikation mit dem Sprachbot wäre einfacher gewesen, wenn …

Freitext…

S e i t e 1 | 1
